# Otology

**Published:** 1892-12

**Authors:** W. H. Harsant


### OTOLOGY.

Dr. Lœwenberg gives the result of his experience of cases of otitis accompanying influenza, observed in Paris during 1891.[2] The affection usually met with was a subacute inflammation of the middle ear, which presented somewhat different phases in different parts of the country. In some places it was accompanied with hemorrhagic myringitis, but in Paris the author only met with a single case of this variety. He did not find it necessary to trephine the mastoid antrum in any of his cases, a fact which he attributes to two reasons: first, in the large proportion of cases, mastoid complications were slight; and second, he first exhausted all mild therapeutic measures, such as large incisions into the drum membrane, inflations of air, and a rigid antiseptic medication, before resorting to trepanning.

He particularly alludes to two peculiar classes of cases: first, some cases which were instantly cured by the air douche; and second, some which were accompanied by a peculiar form of perforation of the drum membrane. In the first class of cases, the patients were mostly children who were suddenly seized with pain in one ear—rarely in both. There was at the same time a sensation of heat and a fullness in the ear. Some fever accompanied these symptoms. Examination invariably showed a drum membrane of a dark red colour, and convex above and behind. A single inflation of air sufficed to remove all the symptoms. Politzer's method was used in children, but the Eustachian catheter in adults. In the second class of cases (of which he records two examples), the inflammation rapidly went on to suppuration, and produced a peculiar pear-shaped perforation of the drum membrane, which the author considers pathognomonic.

[2] *L'Otite Grippale*, 1892.

This was accompanied in one case by a prolonged sanguinolent discharge, which resisted all treatment for a time, but which eventually yielded to mild antiseptic injections, inflations of air, insufflation of dry boric acid, and instillation of alcoholic solutions of the latter.

Dr. William Milligan furnishes another contribution to the now well-known subject of the etiology and treatment of perforations of Shrapnell's membrane occurring in purulent inflammation of the middle ear.[1] He defines Shrapnell's membrane as that part of the membrana tympani lying above the level of the short process of the malleus. Anatomically it differs from other portions of the tympanic membrane in possessing no middle layer of fibrous tissue, hence it is less resistant, and has accordingly been named the *membrana flaccida*. The existence of a minute foramen in this portion of the tympanic membrane has been affirmed by Rivinus, Politzer, Gruber, and others, but denied by Hyrtl and Kessel. The author has found suppuration of the attic, that is, the portion of the tympanum just within Shrapnell's membrane, in five per cent. of the cases of middle-ear suppuration he met with during the last two years. He calls attention to two important points in connection with its etiology. In the first place, it is a curious fact that suppuration of the tympanic attic, accompanied by perforation of Shrapnell's membrane, is hardly ever found as an acute condition. In the second place, it is strange that extensive pathological changes should take place in this portion of the middle ear without a general inflammatory condition of other portions of the tympanic mucous membrane existing at the same time. In almost all the author's cases the disease was secondary to pharyngeal or naso-pharyngeal trouble. In all probability the whole tympanic cavity is affected at first, but later on, owing either to the results of treatment, or to the *vis medicatrix naturæ*, the inflammation subsides in the lower portions of the middle ear, where free drainage is so much more readily obtained; whereas, on the other hand, pus gets locked up in the folds of mucous membrane in this upper segment, and so gives rise to a chronic inflammatory condition of the mucous membrane, which it is exceedingly difficult to eradicate. Adenoid growths are frequently associated with suppuration in this region. Children seem especially liable to it. Caries of the ossicles is a frequent result. Of the three ossicles, the incus is the one most frequently found diseased.

[1] *Med. Chron.*, Oct., 1892.

The treatment of the disease is at times most difficult, and is always tedious. The main guiding principle should be the securing of thorough drainage. The ordinary ear syringe, applied through the external meatus, is not of any great service in dislodging the matter, as the stream of fluid is frequently quite unable to gain entrance through the small perforation. Siegle's pneumatic speculum may be employed with advantage to suck out purulent matter. When the opening is very small, it should be enlarged. The author's intra-tympanic syringe is recommended. Instillations of peroxide of hydrogen (two or three drachms to an ounce) are of much service in penetrating into regions hardly accessible to any form of syringe. In severe cases, where the ossicles are diseased, resort may be had to scraping the diseased area of bone with fine Volkmann's spoons, or to excision of the diseased ossicle or ossicles.

W. H. HARSANT.

---

